# Physiological and Behavioral Mechanisms of Thermoregulation in Mammals

**DOI:** 10.3390/ani11061733

**Published:** 2021-06-10

**Authors:** Daniel Mota-Rojas, Cristiane Gonçalves Titto, Agustín Orihuela, Julio Martínez-Burnes, Jocelyn Gómez-Prado, Fabiola Torres-Bernal, Karla Flores-Padilla, Verónica Carvajal-de la Fuente, Dehua Wang

**Affiliations:** 1 Neurophysiology, Behavior and Animal Welfare Assessment, DPAA, Universidad Autónoma Metropolitana (UAM), Unidad Xochimilco, Mexico City 04960, Mexico; jocelyn.gomez.ilp@gmail.com (J.G.-P.); fabitorber19@gmail.com (F.T.-B.); kkingsleigh@gmail.com (K.F.-P.); 2 Laboratório de Biometeorologia e Etologia, FZEA-USP, Faculdade de Zootecnia e Engenharia de Alimentos, Universidade de São Paulo, Pirassununga 13635-900, SP, Brazil; 3 Facultad de Ciencias Agropecuarias, Universidad Autónoma del Estado de Morelos, Cuernavaca 62209, Mexico; aorihuela@uaem.mx; 4 Animal Health Group, Facultad de Medicina Veterinaria y Zootecnia, Universidad Autónoma de Tamaulipas, Victoria City 87000, Mexico; jmburnes@docentes.uat.edu.mx (J.M.-B.); vcarvajal@docentes.uat.edu.mx (V.C.-d.l.F.); 5 State Key Laboratory of Integrated Management of Pests Insects and Rodents, Institute of Zoology, Chinese Academy of Sciences, Beijing 100101, China; wangdh@ioz.ac.cn

**Keywords:** thermal biology, thermoregulating behaviors, temperature modulation, cutaneous circulation, vascular microcirculation

## Abstract

**Simple Summary:**

The study of the hypothalamic neuromodulation of thermoregulation offers broad areas of opportunity with practical applications that are currently being strengthened by the availability of efficacious tools like infrared thermography (IRT). This review analyzes the effect of climate change on behavior and productivity; and the effects of exercise on animals involved in sporting activities; identifies the microvascular changes that occur in response to fear, pleasure, pain, and other situations that induce stress in animals; and examines thermoregulating behaviors.

**Abstract:**

This review analyzes the main anatomical structures and neural pathways that allow the generation of autonomous and behavioral mechanisms that regulate body heat in mammals. The study of the hypothalamic neuromodulation of thermoregulation offers broad areas of opportunity with practical applications that are currently being strengthened by the availability of efficacious tools like infrared thermography (IRT). These areas could include the following: understanding the effect of climate change on behavior and productivity; analyzing the effects of exercise on animals involved in sporting activities; identifying the microvascular changes that occur in response to fear, pleasure, pain, and other situations that induce stress in animals; and examining thermoregulating behaviors. This research could contribute substantially to understanding the drastic modification of environments that have severe consequences for animals, such as loss of appetite, low productivity, neonatal hypothermia, and thermal shock, among others. Current knowledge of these physiological processes and complex anatomical structures, like the nervous systems and their close relation to mechanisms of thermoregulation, is still limited. The results of studies in fields like evolutionary neuroscience of thermoregulation show that we cannot yet objectively explain even processes that on the surface seem simple, including behavioral changes and the pathways and connections that trigger mechanisms like vasodilatation and panting. In addition, there is a need to clarify the connection between emotions and thermoregulation that increases the chances of survival of some organisms. An increasingly precise understanding of thermoregulation will allow us to design and apply practical methods in fields like animal science and clinical medicine without compromising levels of animal welfare. The results obtained should not only increase the chances of survival but also improve quality of life and animal production.

## 1. Introduction

Living beings have developed various adaptive mechanisms for the many alterations their environment may undergo depending on the place, time, or season in question. These mechanisms include morphological, physiological, and behavioral changes that allow them to confront variable conditions and, in this way, regulate their physiological capacities [1,2,3,4].

On the other hand, adaptation is a relative concept, as premises a set of specific small changes that combine to maintain homeostasis [5]. Examples could include the long, highly-vascularized ears of *Oryctolagus cuniculus* for dissipating heat, the development of thermogenesis from brown adipose tissue (BAT) in placental (eutherian) mammals, the reduced thickness of subcutaneous fat in ruminants from arid regions [6], and the huddling of seals as temperatures decrease [7,8,9]. Mammals have two broad types of thermoregulating mechanisms: physiological (or reflex) and behavioral. Physiological mechanisms consist of involuntary effectors that produce mostly automatic responses that generate or dissipate heat upon the activation of thermoreceptors and the arrival of information to the hypothalamus, relayed through the spinal cord and midbrain. It is essential to understand that the principle thermo-effector tissues include the cutaneous blood vessels, since (i) many metabolic sources of heat (liver, heart, limb muscles) are distant from the skin, where heat is lost; and (ii) body tissues are poor conductors [10]. Some species have specialized thermoregulating organs, like the rat’s tail, that dissipate heat quickly due to its large surface area and dense vascularization [8,11,12]. Studies have also demonstrated that some animals have developed the capacity to induce a hypothermic and hypermetabolic state similar to hibernation to adapt to hostile environments without presenting secondary physiological effects [13].

Behavioral thermoregulation, in contrast, depends on voluntary decisions. Like what occurs with the physiological mechanisms, thermal stimuli are detected by the afferent pathway that transfers the message to the spinal cord and cerebral cortex, influencing the level of perceived thermal comfort and the individual’s decision to gain or lose heat. These thermoregulating behaviors entail goal-oriented actions learned through reinforcement, as was demonstrated long ago [14,15]. One of the most basic thermoregulating behaviors consists of searching out cold or hot habitats that allow the organism to alter its rate of heat loss or gain. In contrast, the most complex thermoregulating behaviors include making nests or burrows [7], social behaviors like huddling with conspecifics [16], and human behaviors like wearing clothes or turning on an air conditioner [8].

In mammals, conserving a relatively constant core body temperature (37 °C in most species) has been essential in maintaining an optimal body system functionality and the sensitive chemical and physical processes involved. Temperatures above 45 °C can cause fatal brain injuries, while those below 27–29 °C can cause cardiac fibrillation, a progressive decrease in respiratory rate, and even death [1,10].

Scientific knowledge indicates that controlling the body’s core temperature is essential for survival [11,17]. Under conditions where thermoregulating mechanisms cannot re-establish body temperature, individuals suffer thermal stress [18]. Observation of the offspring of meerkats (*Suricata suricatta*), for example, a species that inhabits arid regions, has revealed reduced growth and survival rates when ambient temperatures are high [19].

In areas that produce food of animal origin for human consumption, temperature also plays an essential role because poultry, swine, and cattle are all particularly vulnerable due to their high metabolic and growth rates and elevated production levels. Stress caused by intense heat before slaughtering [20], for instance, stimulates muscle glycogenolysis that results in pale, soft exudative meat (PSE) characterized by a low water-holding capacity (WHC). In contrast, stress due to chronic heat reduces the animal’s muscle glycogen reserves, resulting in dark, firm, and dry (DFD) meat with high pH and WHC [21,22,23]. There are also reports on production rates due to the effects of temperature, as in the case of the water buffalo (*Bubalus bubalis*), which, during periods of high ambient temperatures, have been shown to reduce their milk production, growth, and fertility rates due to an inhibition effect on the enzymatic activity generated by extreme heat [24,25,26,27,28,29,30,31].

Infrared thermography is a technique used in both veterinary and human medicine to quantify the skin’s surface temperature based on visualizations of thermographic changes [26,28]. Since thermal stress affects the welfare and productivity of animals [27,28,31,32,33,34,35], studies designed to achieve a complete understanding of thermoregulating mechanisms using tools like infrared thermography (IRT) [25,26,27,28,29,35,36,37] would allow us to comprehend the effects that factors like climate change has on different species.

Thermography can detect the release of surface heat in the form of thermographic images. Thus IRT detects the peripheral microcirculation controlled by the autonomous nervous system, whose parasympathetic element causes the peripheral vasoconstriction of the capillaries nearest the skin, produced by the neurosecretion of catecholamines [36,37]. At the same time, affecting the temperature in situations that are stressful for animals [28] are factors such as post-birth hypothermia [38,39,40], weaning [41] before slaughter [21,35,42], or such surgical procedures as castration [43], and the effect of necessary pre-operatory procedures, including anesthesia [37].

Negative handling in farm animals (shouting and hitting) leads to poor animal welfare, more fear, acute and chronic stress, fear reactions being the most immediate responses that animals show to potentially dangerous stimuli in their environment [44,45,46]. Having mentioned the potential scope and implications of neurophysiological and behavioral studies of thermal responses, the data obtained in scientific studies will help propose solutions to problems related to thermal stress, fear, and other situations that threaten the health and welfare of domestic and wild animals [27,28,29,30,47,48,49,50]. This review, therefore, analyzes the main anatomical structures and neural pathways that allow the generation of autonomous and behavioral mechanisms that regulate body heat in mammals, information that can be used in related disciplines and areas of opportunity.

## 2. The Skin’s Role in Thermoregulation

### 2.1. Reception of Thermal Responses

**Figure 1:**
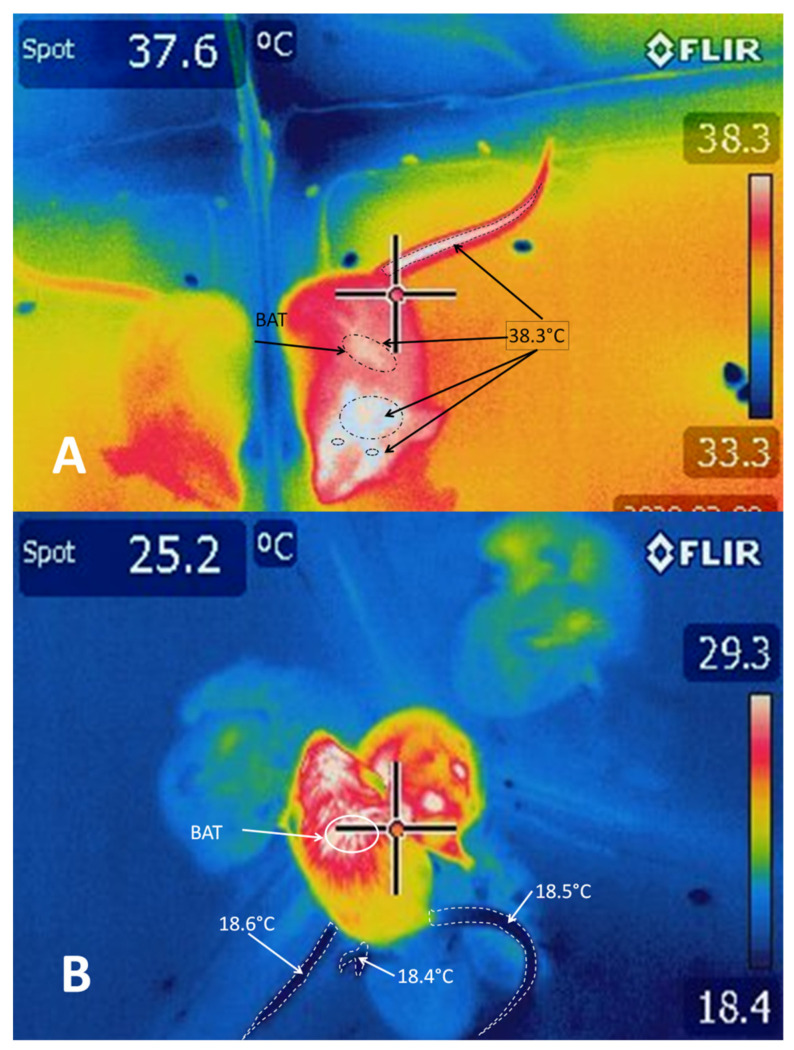
Thermograms of mole-rats subjected to (**A**) heat and (**B**) cold. Clearly, under hot ambient temperatures, the body’s entire surface presents a temperature above 33.3 °C, with a maximum of 38.3 °C on the face, back, and tail. In contrast, in a cold environment (**B**), the tail and pelvic limbs present a surface temperature of 18.4–18.6 °C, with a maximum of 29.3 °C in the head, eyes, and back. Observations show that heat distribution differs depending on the stimulus. In warmer environments, the zones with greater heat are the eyes, head, back (the prescapular zone where brown adipose tissue (BAT) is stored), and tail; while under cold conditions, heat concentrates in the eyes, head, and the first part of the back. Thermogram A shows the mechanism of peripheral cutaneous vasodilation under high temperatures, which increases heat transfer towards the environment, while thermogram B illustrates the mechanism of peripheral cutaneous vasoconstriction, visible on the pedal surfaces and tail, at low temperatures. This mechanism conserves heat in the organism’s core region to protect its vital organs [54]. The images also show that the tail and feet are key organs for conserving and dissipating heat in two thermally distinct environments. These findings agree with McGowan et al.’s report on mole-rats [53]. The animals shown in the thermograms were subjected to ambient temperature changes for only 30 min, following Mexican regulations for the care and use of experimental animals. (Images obtained with an FLIR E 50 camera equipped with an 18-mm FOL lens at a resolution of 240 × 180 pixels, (emissivity = 0.95, distance = 0.5 m)).

The skin and its components (fur, wool, hair, glands, and nerve endings like Merkel’s disc receptors and hair follicles) [51] form one of the main organs that detect external chemicals or physical agents to which mammals may be exposed. The skin initiates protective measures in response to changes in temperature, solar radiation, and humidity, among many others. Adaptations of this organ have led to the development of mechanisms that help to deal with alterations that may constitute active threats. These mechanisms include preventing water loss and extracellular liquid through the tallow secreted by sebaceous glands when the temperature is high [52]. For example, when McGowan et al. [53] evaluated differences in thermoregulation by analyzing the corporeal region of various species of African mole-rats taken from different habitats and subjecting them to changes in ambient temperature, they found that the core body temperature of mole-rats (*Cryptomys hottentotus natalensis*) from Natal increased significantly at ambient temperatures >24.5 °C. However, rats living in high altitude areas (*Cryptomys hottentotus pretoriae)* (savannah) and Damaraland (*Fukomys damarensis*) (arid/semi-arid) had core body temperatures within a narrower range. One particularly significant finding was that the surface temperature of the Natal rats was regulated mainly by pedal surfaces (Figure 1, thermograms of mole-rats), while in the Damaraland rats, the ventral surface was the central region that dissipated excess heat.

The detection of heat changes is performed by nerve endings with transient receptor potential (TRP), specifically, type 8 [55,56], functioning as cation channels. Typical physiological responses to cold environments include shivering and cutaneous vasoconstriction (CVC), which prevent heat loss and generate heat. In contrast, under high temperatures, the primary somatosensory neurons in the skin are activated by type-2 TRP stimulation. Studies have demonstrated that this type of stimulation is not present in mice; thus, their behavioral thermoregulating response is compromised [57]. All the afferences that the skin receives carry information to an integrating center in the central nervous system (CNS), which generates a response designed to maintain the various enzymatic, metabolic, reproductive, behavioral, and other systems involved in temperature regulation. This integrating system is explained in detail below [58].

### 2.2. Temperature Modulation through Cutaneous Circulation

**Figure 2:**
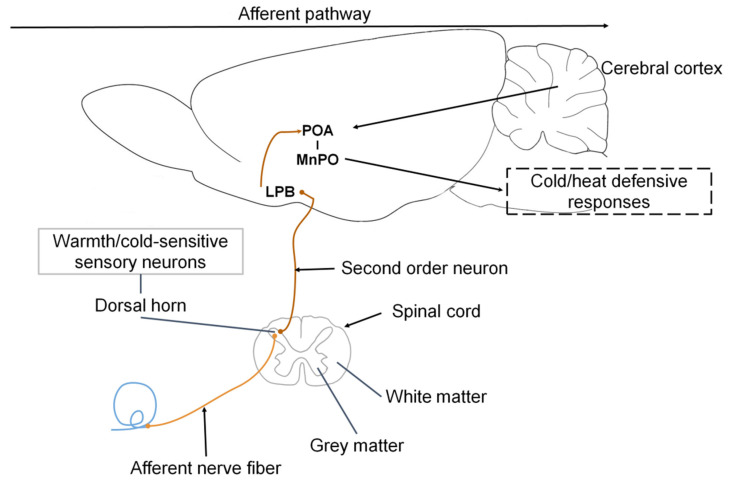
General mechanism of physiological thermoregulation in mammals. This process begins when the organism’s thermoreceptors in the skin detect a temperature change, either environmental or internal. Upon detecting the new temperature, those thermoreceptors transmit a signal through afferent nerve fibers to the dorsal horn of the spinal cord, where warm/cold-sensitive sensory neurons are activated. These neurons, in turn, transmit a response that consists of a nerve impulse sent to the lateral parabrachial nucleus (LPB) [63]. This triggers a reaction in the preoptic area (POA) of the hypothalamus—a structure considered responsible for thermoregulation in mammals [64]—specifically, the median preoptic nucleus (MnPO) [8]. After that, cold- or heat-defensive responses are initiated through efferent pathways [1].

The different layers of the skin have distinct adaptations to mediate heat fluctuations. Plotczyk and Higgins [59] mention that the extensive vascular network of the dermis is mediated by numerous endothelial cells, smooth muscle, and synapses of groups of somatosensorial neurons (proprioceptors, mechanoreceptors, nociceptors, thermoreceptors) [60] of the dorsal horn of the spinal cord and the spinal trigeminal nucleus that respond to innocuous cooling or heating of the skin [46,61] (Figure 2). This vast network runs into the surface and deep plexuses; the former is characterized by capillary circulation, where the participation of the papillary loops is visible. The deep plexuses consist of blood vessels of a larger diameter that reach the hair follicles and conducts of sebaceous and sweat glands [62].

Cutaneous vasomotor adjustments form part of the effector changes that maintain thermal comfort, mediating the transfer of heat by convection between the body’s core and the skin’s surface through the bloodstream [12]; for example, when an increase in body heat occurs in humans, the magnitude of skin vasodilatation may reach 6–8 L/min during hyperthermia [65].

The need to increase or reduce heat dissipation depends on environmental factors. It is related to reflex control at the neural level, with the aid of two branches of the sympathetic nervous system (SNS): a noradrenergic system that mediates vasoconstriction and a cholinergic system that causes vasodilatation [66].

It is important to note that autonomous thermoregulating responses, in addition to producing or dissipating heat through cutaneous vasomotor responses, foster heat generation via tremor and non-tremor mechanisms [67]. These processes are described below.

#### Temperature Modulation Mediated by Sweating

Another mechanism used to dissipate heat is sweating, whose primary function is to thermoregulate by evaporation. There are two types of perspiration: insensitive, the constant loss of heat that is not detected by sight or touch, and sensitive, which can be observed, as when humans exercise [51]. Sweating maintains stable body temperature and functions to excrete substances like urea, uric acid, ammonia, and lactic acid, among others [68]. We discuss this neurophysiological response mechanism in greater detail below.

In summary, the thermoregulating capacity of the skin and its modulation through the SNS through effector changes and the scope of vascularization in this organ depend on the different thermal and dietetic stimuli involved in determining whether the body needs to dissipate or preserve heat.

## 3. Hypothalamic Control of Deep Body Temperature

The control of heat to maintain a constant deep body temperature is a characteristic of most mammals and one closely related to the integrity of specific CNS structures that converge in a group of common neurons that act to keep the deep temperature of the animals stable [51]. The core body temperature is regulated primarily through a negative feedback circuit. A sensor monitors the temperature, activated when a change is detected, and corrective action will occur in proportion to the magnitude of the disturbance [69,70]. One of the key sites where core temperature is monitored is the preoptic area (POA) of the hypothalamus, which contains heat thermosensitive neurons representing 30% of all the neurons in this area [17]. Their activation increases as the local temperature increases and decreases as the POA temperature falls. The neurons in this area have *Ptgds*; which are genetic markers that codify an enzyme, which synthetizes prostaglandin D2 (PGD2).

In response to a decrease in temperature, the activity of these neurons increases PGD2 production, thus activating the DP1 receptor and exciting postsynaptic neurons. When the stimulus reaches the pancreatic β cells, insulin secretion is adjusted to raise the levels and sensitivity to this hormone in the receptors, in addition to a secure and adequate supply of glucose for thermogenic tissues [71]. Other receptors that participate in the regulation and sensitization of the POA are called leptin receptors, which regulate the thermogenesis of brown adipose tissue and control reproductive functions [72].

Neurons in the medial ventral POA (vMAP) express the brain-derived neurotrophic factor (BDNF), as do GABAergic neurons in the lateral ventral POA (vLPO) [57,73], which have a close relation to heat gain, by activating basic metabolic functions.

A study by Song et al. [74] found that transient receptor potential cation channels (TRPM2) form part of a large family of transient thermosensitive reception channels (TRPV1, TRPV2, TRPV3, TRPV4, TRPM2, TRPM3, TRPM4, TRPM5, TRPM8, TRPA1) that are specific to mammals. These channels detect the surface temperature of the neurons of the POA. It is important to note that the functional expression of the Thermo-TRPs is evident in sensory neurons, tissues, and cells, that are not exposed to drastic temperature changes [75] but are essential for sensitivity and participation in detecting heat stress and modulation of body temperature during a fever.

Schlader et al. [76] suggested that the onset of thermoregulatory behavior in humans during exposure to hot or cold environments is not preceded by acute increases in sweat rate or the rate of metabolic heat production. The onset of thermoregulatory behavior is preceded by acute changes in vasomotor tone in hairless skin (non-glabrous skin), contrasting with the stability of vasomotor tone in the skin with hair (glabrous skin). Schlader et al. [76] show that sweating or shivering is not required for thermal behavior to start in humans. In the skin, thermoreceptors are the nerve endings of Aβ, Aδ (myelinated), and C (unmyelinated) neurons. At this level, thermal stimuli cause depolarization in thermoreceptors, generating transduction; consequently, upon reaching the threshold, action potentials are generated where the neuronal terminal buttons release the neurotransmitters that activate the synapses; this process is called transformation. Thermoreceptors perceive the thermal sensation, made up of ionic channels called transient potential receptors (TRP), which detect different temperature ranges (TRPV2, TRPV3, TRPV4, TRPM8, and TRPA1) [8,77].

At the level of the hypothalamic receptors, Kamm and Siemens [78], in a mouse model, demonstrated that the TRPM2 ion channels located in the POA neurons function as heat sensors registering temperatures above 37 °C as demonstrated through electrophysiological tests. Furthermore, it has been shown that increased activity (excitation) of TRPM2 WSN (warm sensitive neurons) favors heat loss leading to hypothermia; the evaluation of temperature and the rats’ tail vasodilation degree was part of the animal model studied. On the contrary, the inhibition of TRPM2 neurons promotes the increase in Tcore until hyperthermia. Stimulation of thermoreceptors and their TRPM2 ion channels can be useful in the study of experimental and clinical hypothermia [74]. Thus, it has also been shown that treatment with TRPM8 agonists (cold response) causes hyperthermia, while TRPM8 antagonists cause hypothermia [8]. On the other hand, it is also proposed that the TRPV1 and TRPM2 channels function as heat sensors in the brain. In TRPV1, central injection of the TRPV1 agonist capsaicin induces hypothermia [79].

The neurons of the medial preoptic area (MPO) can be stimulated by hypothalamic temperature and temperature changes in the spinal cord, blood (the MPO is highly-perfused), and viscera (esophagus, stomach, large intra-abdominal veins, and mesentery, among others) that receive thermoreceptive afferent signals through the vagus nerve. This nerve sends information to the POA zone through the nucleus of the solitary tract in the caudal portion of the medulla oblongata, which is the termination and projection site of the cardiovascular and visceral afferent fibers of the glossopharyngeal and vagus nerves towards the CNS [64,80]. The information sent over these pathways is projected through neural connections to the hypothalamus to produce an adequate response.

In summary, POA neurons form a convergence point, which integrates information about the temperature of all corporeal regions, including the periphery of the animal. It is a point where the various cellular activities, tissues, and effector organs capable of dissipating or conserving heat, are managed and controlled.

## 4. Neurophysiological Responses for Controlling Hyperthermia

Under normal resting conditions, the body generates and dissipates heat to maintain temperature stability. When the speed of either mechanism is subtly altered by changes in the environment or in the organism itself, this imbalance induces a state of hyperthermia or hypothermia [25,31,34,39,40,46,51,81]. A human body temperature above 104 °F (40 °C) is defined as severe hyperthermia; that is, a state of high temperature that, in normothermic organisms, triggers mechanisms as cutaneous vasodilation and eccrine sweating or evaporative cooling (conduction and convection), which constitute the principal means of modulating such alterations [58]. Body heat is significantly regulated by the central nervous system (CNS), which receives, deciphers, and sends signals through diverse structures to activate the mechanisms that manage heat dissipation. Here, the skin functions as a target organ by perceiving external and internal temperature changes and relaying them to the CNS in the form of afferent stimuli [82]. Before the 1990s, the POA was reported as the structure of the CNS responsible for thermoregulation in mammals. The CNS is in the anterior area of the hypothalamus (anterior preoptic hypothalamus) [83] and receives information through stimuli perceived by the thermoreceptors distributed in the center and periphery, monitoring the blood temperature and skin, respectively. Once the POA integrates those signals, it sends them through the brainstem to the heat loss center to activate sudomotor and vasomotor nerves and exert negative feedback on heat-promoting centers [51,82]. In some studies, the POA has been stimulated directly with heat to demonstrate its importance in thermoregulation. Results showed cutaneous vasodilatation, panting, sweating, and changes in the behavior of diverse animals [58,84].

The skin has a complex, sympathetic innervation that includes several nervous structures: vasodilators, vasoconstrictors, sudomotors, pilomotors, sensory fibers, or thermoreceptors [82]. For this reason, it is a vital organ for regulating hyperthermia. Thanks to its structures, the skin can detect changes in ambient temperature and transmit the perceived stimuli directly to the POA, permitting the generation of effective responses to defend the organism’s thermal homeostasis [64]. In addition, its nervous structures receive nervous stimuli sent by the heat-loss center to activate mechanisms like sweating and vasodilatation [70]. It is important to note that the activation of all these mechanisms is controlled by the release of diverse chemical substances that function as messengers inside the communication network called the nervous system [82].

In a general description, the POA contains warm-sensitive neurons that, upon perceiving an alteration, send a stimulus to the sympathetic preganglionic neurons distributed in the nucleus of the intermediolateral area of the spinal cord, which connects to the sympathetic ganglia responsible for innervating the blood vessels, cutaneous vessels and diverse glandular structures that participate in modulating hyperthermia [1]. Similarly, the information perceived by the cutaneous thermoreceptors is transmitted through synapses to the dorsal horn and trigeminal nerve, and from there is relayed to the lateral parabrachial nucleus, which connects to various nervous regions connected to the POA [1].

Nowadays, the mechanism that allows the integration of the information captured by the cutaneous thermoreceptors remains unknown. As mentioned above, organisms use two main mechanisms to control hyperthermia: cutaneous vasodilatation and evaporative cooling.

### 4.1. Cutaneous Vasodilatation

**Figure 3:**
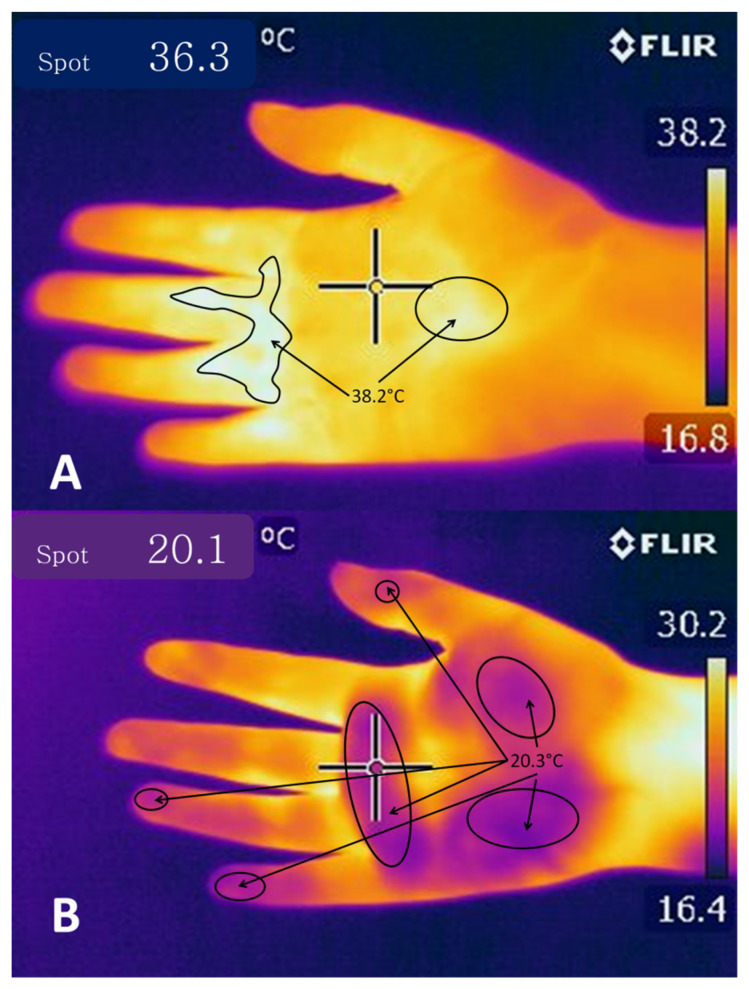
Thermograms of a hand subjected to (**A**) heat and (**B**) cold for 30 s. These images show that in response to a hot stimulus, a larger area of the palm presents a temperature of 36.3 °C with a maximum of 38.2 °C in the center and around the proximal and distal transverse creases. In contrast, in response to a cold stimulus, most of the palm—marked with ellipses—and the fingers present a temperature around 20.3 °C, while only the wrist, the center of the palm, and a small area of interdigital spaces have a maximum of 30.2 °C. Observations show that heat is distributed across almost the entire surface of the palm in response to a hot stimulus due to heat-induced vasodilatation. In response to a cold stimulus, in contrast, the surface temperature decreases, and heat concentrates in strategic points due to peripheral vasoconstriction. (Images obtained with a FLIR E 50 camera with an 18-mm FOL lens at a resolution of 240 × 180 pixels, (emissivity = 0.95, distance = 0.5 m)).

The function of cutaneous vasodilatation is to divert central circulation into peripheral circulation to dissipate heat using blood as a vehicle. Nowadays, two mechanisms of vasodilatation are known: active vasodilation, characterized by augmenting blood flow by increasing nervous activity, and passive vasodilation, which also increases blood flow, but by reducing the activity of the vasoconstrictor nerve [82,85] (Figure 3). Some studies speculate on control through a network of active neurogenic vasodilators in specific sites of the glabrous skin [86]. Vasodilatation is induced when the POA receives excitation or warming stimuli. We know that two populations of nerve cells that regulate vasomotor responses are in the caudolateral POA [87]. As with the mechanisms for controlling hypothermia, the rostral raphe pallidus and rostral ventrolateral medulla (RVLM) participate in inducing vasodilatation. However, it is proposed that the mechanism is induced by inhibiting these zones into block vasoconstriction [88].

**Figure 4:**
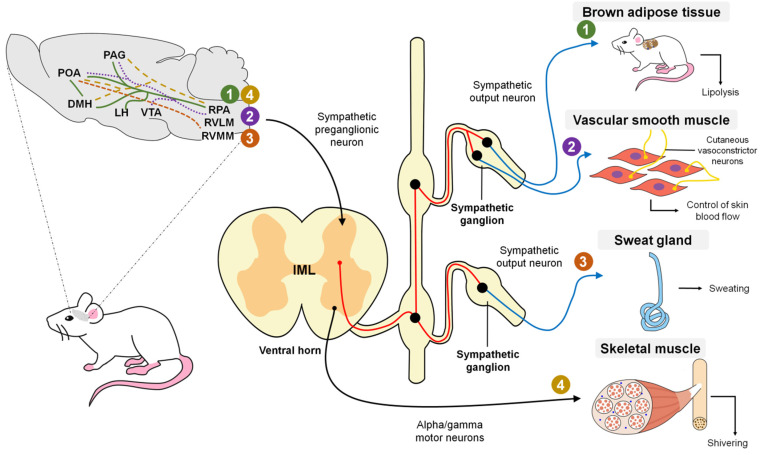
Efferent thermogenic pathways in mammals. Green (marked with the number 1) represents the efferent pathway for BAT (brown adipose tissue) thermogenesis; purple (marked with the number 2) indicates the efferent pathway that produces changes in the skin blood flow; orange (marked with the number 3) shows the efferent pathway for the production of sweat; yellow (marked with the number 4) exhibits the efferent pathway for shivering thermogenesis. Red lines show the path that occurs in the sympathetic preganglionic neurons, while the blue lines indicate the path that takes place on the postganglionic neurons. The broken line (-----) indicates that the anatomical pathway that connects said structures is unknown. Preoptic area (POA); dorsomedial hypothalamus (DMH); lateral hypothalamus (LH); periaqueductal gray (PAG); ventral tegmental area (VTA); raphe pallidus (RPA); rostral ventrolateral medulla (RVLM); rostral ventromedial medulla (RVMM); intermediolateral column (IML). (Exclusive images created by Adriana Domínguez).

Regarding vasodilatation, it is believed that the dorsomedial hypothalamic nucleus (DHM) is not required for cooling since the probability of direct projections between the POA and rostral raphe pallidus (rRPA) has already been mentioned [57] even though the ventral tegmental area (VTA) and rostroventrolateral PAG have been posited as brain regions that substitute the chemical stimulus to the rRPA from the DHM [89] (Figure 4).

It is not clear whether, as in hypothermia, the population of glutamatergic cells is responsible for inducing heat-dissipating mechanisms since some studies have described the participation of GABAergic cells [73]. However, the participation of any of these cell populations is possible as evidence indicates that a large portion of the POA neurons express for these two receptors. In addition, since these two populations are heterogeneous, it is necessary to identify objective molecular targets that will enable better understanding of the efferent pathways that activate the associated mechanisms [8].

### 4.2. Evaporative Cooling

Evaporative cooling occurs mainly through sweating. In non-human mammals, other mechanisms may cool by evaporation with species-specific variations; examples include salivation and spreading saliva over the entire cutaneous surface or fur and panting performed through the respiratory tract [64]. A comparative study carried out in 1998 of normothermic vs. hyperthermic conditions set out to determine the sympathetic activity characteristic of these states. Results showed that sweating activity increased by 80% in the hyperthermal state, reflecting its predominance in that condition; in other words, evaporative cooling constituted the main mechanism of heat dissipation [90]. Sweating is also considered an important thermo effector in humans. This process occurs through the eccrine glands, which consist of a spiral secretor in the form of a spiral-shaped, tubular gland that contains a secreting and a proximal conductor located on the dermis, where multiple nervous fibers are enveloped by a dense network of capillaries are found [91]. Accompanied by a group of other cell types, these fibers respond to hydrostatic pressure by generating sweat [92].

It has been determined that sweating in rodents is mediated by the release of acetylcholine, a chemical mediator in the synaptic areas of the sympathetic nerves located in the peripheral sweat glands [8]. Specifically, sympathetic innervation is stimulated by a package of nerves that terminates in the preganglionic neurons residing in the intermediolateral cells of the spinal cord.

The column of IML cells has projections to the rostral ventromedial medulla (RVMM), which correlates to sweating in cats and humans [93]. Currently, the stimulation pathways between the RVMM and POA are not entirely known, so they are still under study [8] (Figure 4).

There is a vast field of research concerning the evaporation mechanisms based on salivation and panting because the pathways through which these mechanisms are stimulated remain unclear. We assume that they are mediated autonomously by the secretion of saliva and liquid in the respiratory tract, by a somatic component that induces saliva-producing behavior—explained below—and increased airflow through the upper respiratory tract [64]. An important finding is that the mechanism of evaporation demands a high caloric cost for the organism and entails significant physiological alterations, such as excessive water loss that alters osmotic stability. As a result, food must be available to cover the caloric and water costs and stabilize this balance. An option is high caloric density diets so that the individual can replenish energy losses with a small amount of food. Nonetheless, in species that inhabit arid regions during seasons marked by a drastic shortage of food and water, there are reports of adaptations that allow a considerable temperature increase that may induce a state of hyperthermia which reduces energy costs and water loss by up to 50%. This adaptive process may benefit from the recent substantial increase in ambient temperatures worldwide [94].

Another alteration caused by states of acute hyperthermia is hypoxia, which affects mainly highly irrigated organs like the intestinal mucosa, producing tissue damage that results in increased permeability [95]. For this reason, intensive production systems have been forced to design methods to reduce the impact of heat stress on their animals [96].

Although the cooling mechanisms of vasodilatation and evaporation are described in the literature individually, and it is believed that distinct nervous circuits mediate them, they are broadly related because vasodilatation provides heat and blood plasma; that is, the resources used as the fluid required for evaporation to occur through sweating, salivation, and panting [58]. Studies have also documented that when sweat and salivary glands are activated, they release an enzyme that catalyzes bradykinin, a peptide with a high capacity for stimulating vasodilatation. When released into the interstitial space, bradykinin causes active vasodilatation, suggesting that it is not a mediator of the activation of cutaneous vasodilatation [97].

### 4.3. Response to Exercise

During dynamic exercise, the organism concentrates large quantities of metabolic heat produced by muscular contractions. The body’s deep temperature rises due to the heat generated, so cooling mechanisms like vasodilation and evaporation are activated to dissipate excess heat [98]. These specific mechanisms are considered independent from the changes in deep temperature at the onset of exercise; in other words, sweating and vasodilatation can be mediated by exercise even before the organism registers changes in its deep temperature [99]. It has been observed that the fatigue generated by exercise works as an inhibitory stimulus sent to the hypothalamus in response to hyperthermia, where it causes a decrease in the resistance to exercise that functions as a heat-dissipating mechanism [100].

**Figure 5:**
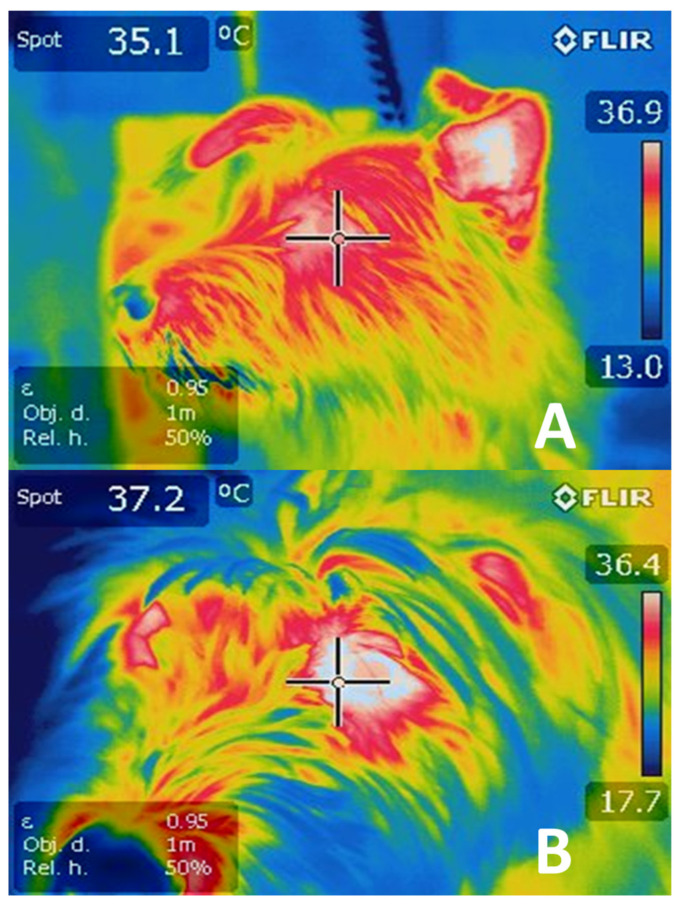
Thermograms of an idle dog (**A**) vs. a dog doing exercise (**B**). During repose (**A**), the ocular temperature was 35.1 °C, compared to image (B showing an ocular temperature of 37.2 °C in a dog after 15 min of exercise. (Obtained with an FLIR E 50 camera with an 18-mm FOL lens at a resolution of 240 × 180 pixels, (emissivity = 0.95, distance = 1 m)).

Tanda [101] carried out a study with runners utilizing IRT that allowed him to achieve his main objective: to relate the concentration of metabolic heat with ambient temperature, genetic traits, training, acclimatization, and skin surface temperature. The main corporeal areas considered in that study were the arms, torso, and legs. Findings showed that the duration and intensity of exercise were involved in the concentration of heat in the body. Depending on the capacity of different bodies to dissipate heat through the methods outlined previously, the runners presented central temperatures from 38.5 to 40 °C at the end of the competitions, whether the distances involved were short or long. The study concluded that skin temperature represents the main variable that controls heat exchange during body-environment interaction and that this is due to changes in blood flow caused by vasoconstriction and vasodilatation in the skin. To appreciate changes in ocular temperature registered by IRT in dogs subjected to exercise, see Figure 5.

Today, it is of great clinical importance to monitor the body temperature of certain mammal species involved in sporting activities [102]. Methods have been created that allow the objective measurement of temperature changes on the body’s surface, including the aforementioned infrared thermography (IRT) technique [36]. Normative thermography data, however, remains incomplete [12]. A pilot study using IRT was conducted in 2017 to demonstrate the changes observed in horses subjected to different times and intensities of physical exercise. All the regions of interest showed an average temperature increase of 2 °C, indicating that IRT is an effective tool, though additional research is required to improve its performance [103].

### 4.4. Fear Response

The fear response is another phenomenon that triggers behavioral and physiological changes through which organisms seek to increase their chances of survival by reacting adequately to threats occurring in the environment [104]. It is not clear how the nervous system participates in the fear response, but it has been suggested that the onset of these reactions and the changes observed as a consequence depend on connections among the thalamus, amygdala, and hypothalamus. The thalamus perceives the environmental threat and sends the information to the amygdala, where it is processed and forwarded to the dorsomedial hypothalamus area (DHM) [105]. It is important to recall that the DHM contains nerve cells to trigger thermogenesis and cutaneous vasoconstriction [106]. It is well-known that the amygdala is a region of neural control involved in the emotions but also plays a vital role in fear conditioning, and it has been noted that it has synaptic connections that endow it with the ability to store memories related to threat stimuli or fear conditioning [107].

**Figure 6:**
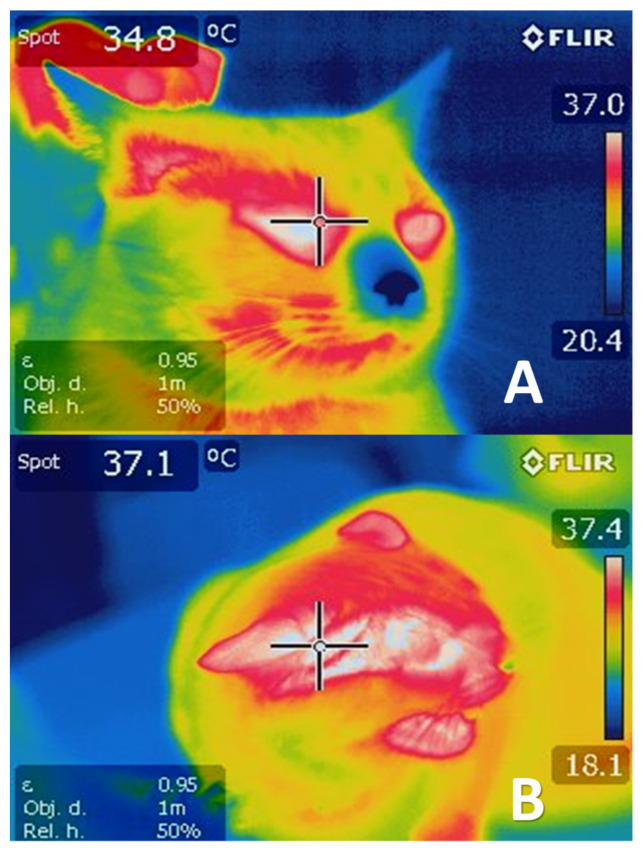
Thermogram of an idle cat (**A**) and one exposed to a predator (**B**). The cat at rest (thermogram (**A**)) has an ocular temperature of 34.8 °C, but the one in thermogram (**B**), which was exposed to a dog for 60 s, shows an ocular and auditory conduct temperature of 37.1 °C. Thus, the ocular temperature increased by around 2 °C in the presence of the adverse stimulus. The cat in this image was exposed to the predator at a distance of 1 m for only 60 s, so no harm or injury occurred. This protocol complies with Mexico’s regulations for the care and use of experimental animals. (Images obtained with an FLIR E50 camera with an 18-mm FOL lens at a resolution of 240 × 180 pixels, (emissivity = 0.95, distance= 1 m)).

Research has shown that when threats trigger fear reactions in rats, these animals undergo various cardiovascular changes involving a decrease in blood perfusion in areas like the tail and limbs, which are considered zones that have a greater capacity to dissipate heat and that in threatening situations are the most exposed parts of the body. The decreased perfusion is due to cutaneous vasoconstriction of these specific areas, perhaps because the rat is preparing for either “fight-or-flight” and so acts to prevent significant blood loss through these vulnerable anatomical structures [104,108]. Vasoconstriction redistributes the blood from the peripheral circulation to the skeletal muscles to ready the rat to fight or escape. This response has been observed under IRT as a concentration of large amounts of heat on the dorsal surface of the rats’ body, and shallow temperatures in the limbs and skin surface since this redistribution of the blood also modifies heat concentrations [108]. In this regard, the development of scientific tools has been oriented toward efforts to reduce their impact on organisms. As a result, recent findings indicate that behavioral, physiological, and emotional modifications associated with stressful stimuli can be measured not only by utilizing such biomarkers as lactate, glucose, and cortisol, among others [109,110], but also by methods like infrared thermography [27,28,29], observations of body language and facial expressions [45,111], and more advanced techniques, such as electroencephalography (EEG) and computed tomography (CT) [27]. These approaches make it possible to obtain values with greater accuracy and objectivity without compromising the animals’ welfare. Figure 6 shows changes in the rostral surface temperature of a cat captured by the IRT technique when it was exposed to a predator.

## 5. Neurophysiological Responses for Controlling Hypothermia

Ascending signals from the thermoreceptors in the skin, viscera, and spinal cord reach the cold-sensitive sensory neurons innervating the superficial laminae of the dorsal horn, which then send glutamatergic projections to the lateral parabrachial nucleus (LPB), specifically the external lateral LPB (LPBel) [8]. The cold-activated LPB neurons send glutamatergic projections to the midline POA, specifically the median preoptic nucleus (MnPO), which, through efferent pathways involving the sympathetic and somatic motor systems, trigger cold-defensive responses, like vasoconstriction and thermogenesis to block heat loss [1].

Madden and Morrison [54] mention that the thermogenic efferent pathway (Figure 4) involves an inhibitory output of the POA that affects the hypothalamic neurons in the dorsomedial hypothalamus. The thermogenesis-promoting neurons of the dorsomedial hypothalamic nucleus (DMH) activate premotor neurons in the raphe pallidus area (RPa) that, in turn, send a descending excitatory drive to the spinal neurons (sympathetic preganglionic neurons for BAT; motor neurons for shivering). In the case of cutaneous vasoconstriction, a similar pathway mediates the process. The difference lies in the fact that no relay in the DMH is required because the inhibitory output of the POA directly impacts the sympathetic premotor cutaneous vasoconstrictor neurons in the raphe. The following is a more detailed explanation of these two processes.

### 5.1. Vasoconstriction

In response to cold, the sympathetic nerve fibers that innervate the cutaneous vasculature are activated, causing cutaneous vasoconstriction that (i) reduces heat transfer with the environment and (ii) conserves heat in the center of the organism’s body [54] (Figure 1). A study with rats by Tanaka et al. [112] indicated that the neurons of the median preoptic nucleus (MnPO) play an essential role by triggering the action of the sympathetic cutaneous vasoconstrictor (CVC) nerve fibers. Those authors observed that inhibiting the MnPO neurons by microinjections of GABA caused vasoconstriction in the tails of mice (*Mus musculus*) due to the increased activity of the sympathetic CVC nerve fibers. The optogenetic activation of the glutamatergic neurons in the MnPO by channelrhodopsin 2 increases vasodilatation in the tail [106,113]. The above suggests that an output from the MnPO inhibits the sympathetic CVC nerve fibers. Excitation of the rostral raphe pallidus (rRPA) also increases vasoconstriction and reduces the cutaneous temperature of the tail (Figure 1), while its inhibition blocks vasoconstriction [8].

**Figure 7:**
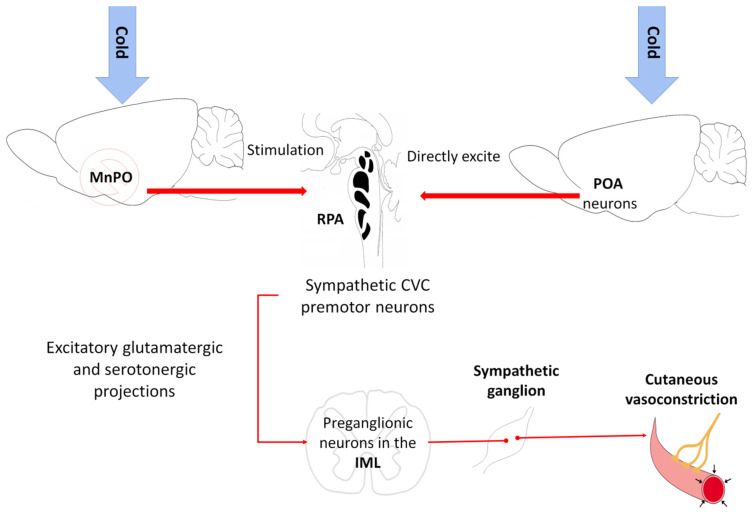
Neurophysical mechanism for developing the cutaneous vasoconstriction response. Perceptions of cold in the brain cause inhibition of the median preoptic nucleus (MnPO), which stimulates the sympathetic cutaneous vasoconstrictor (CVC) premotor neurons in the raphe pallidus area (RPA). These neurons send excitatory glutamatergic and serotoninergic projections to the preganglionic neurons in the intermediolateral cell column (IML) of the spinal cord that, when activated, generate peripheral vasoconstriction. Note that activating the preoptic area (POA) neurons by a cold stimulus can be excited directly through the sympathetic CVC premotor neurons.

Although the raphe pallidus area (RPa) has sympathetic premotor neurons for cutaneous vasoconstriction [114], it appears that the inhibitory output from the MnPO is an indirect input to the sympathetic CVC premotor neurons in the RPa [115,116]. The skin cooling activates neurons in the POA to directly excite the RPa’s CVC premotor neurons by activating glutamatergic receptors [117]. In response, the RPa’s CVC premotor neurons trigger cutaneous vasoconstriction through the excitatory glutamatergic and serotoninergic projections to the preganglionic neurons in the intermediolateral cell column of the spinal cord [54] (Figure 7). It is important to note that some spinally-projecting neurons of the rostral ventrolateral medulla (RVLM) are inhibited by warming the POA [118] and contribute to the activity of the sympathetic nerves of the CVC SNA [119]. However, the RVLM plays only a minor role in CVC activity compared to the RPa’s sympathetic premotor neurons. The neural pathway that transmits thermal information from the POA to this region is unknown.

### 5.2. BAT Thermogenesis

Brown adipose tissue (BAT) is a specialized organ for rapid heat production. In mice, it is found mainly in the interscapular region, an area highly innervated by sympathetic nerves. Tan and Knight [8] affirm that the release of norepinephrine from this sympathetic innervation induces a mitochondrial leak in BAT that consists of a facilitated proton leak through mitochondrial membranes of the brown adipocytes that occurs due to the elevated release of the uncoupling protein-1 (UCP1) [120], producing heat. This process is known as non-shivering or BAT thermogenesis. From a physiological perspective, it is a by-product of the inefficiency of mitochondrial Adenosine triphosphate (ATP) production and its utilization in the skeletal muscle [121]. Observations of this process, at least in rats, show the involvement of such structures as the rostral raphe pallidus (rRPA) [122] and dorsomedial hypothalamic nucleus (DMH) [123], since chemical stimulation in these regions induces BAT thermogenesis. Indeed, direct optogenetic stimulation of the DMH/rRPA projection causes BAT thermogenesis in rats [124]. However, the precise identity and connectivity of these neurons are not yet clear.

In cold environments, the cool-afferent input to the median preoptic nucleus (MnPO) excites its neurons, which in response inhibit the activity of a population of inhibiting neurons in the medial preoptic area (MPA), suppressing activation of the BAT thermogenesis-promoting neurons [54]. In response to both cold stimuli and fever, activation of the BAT thermogenesis-promoting neurons in the DMH is likely due to the elimination of the active thermogenesis-suppressing output and activation of glutamatergic receptors in the DMH neurons [125]. However, activation of a subpopulation of neurons that contain leptin receptors (LepR) has been detected in mice [126,127]. In addition to the DMH, regions such as the arcuate nucleus of the hypothalamus (ARC), also involved in the BAT thermogenesis, have a high expression of LepR as well [128]. Another family of molecules related to BAT is the opsins. One example of these is opsin 5, a photoreceptor group capable of detecting light signals in the environment. These glutamatergic neuropsins in the POA can also be expressed in the retina, skin, adrenal glands, liver, heart, and pancreas [129].

**Figure 8:**
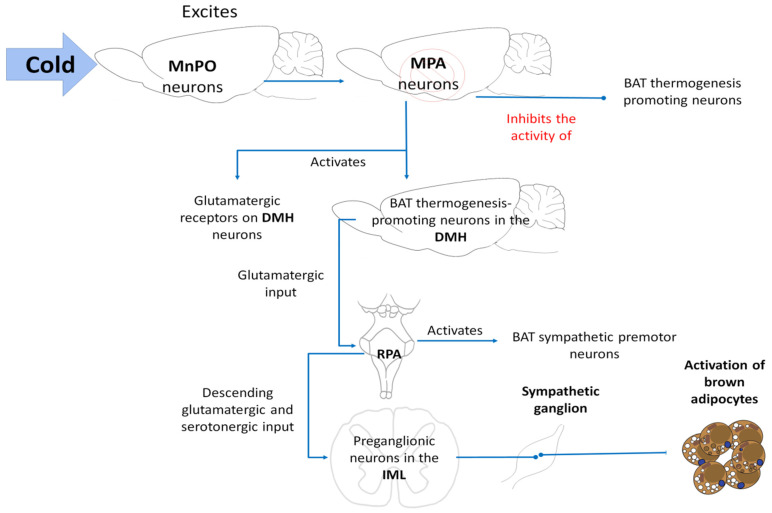
Neurophysiological mechanism for developing the BAT thermogenesis response. Detection of cold excites the neurons of the median preoptic nucleus (MnPO), which inhibits the activity of a group of neurons in the medial preoptic area (MPA) whose function is to suppress the activity of brown adipose tissue (BAT) thermogenesis-promoting neurons. The inhibition of said neurons and excitation of the glutamatergic receptors of the neurons in the dorsomedial hypothalamic nucleus (DMH) activate the BAT thermogenesis-promoting neurons in that structure. These neurons send a glutamatergic signal that activates the BAT sympathetic premotor neurons in the raphe pallidus area (RPA), increasing the descending glutamatergic and serotonergic input to the intermediolateral cell column (IML). These are the substances that trigger BAT activation.

Activation of the BAT sympathetic premotor neurons in the RPa, likely through glutamatergic input from the DMH to the RPa [124,130], promotes the descending glutamatergic and serotonergic input to the spinal cord, since glutamate and serotonin in the spinal cord contribute to activating BAT [54] (Figure 8).

### 5.3. Shivering

**Figure 9:**
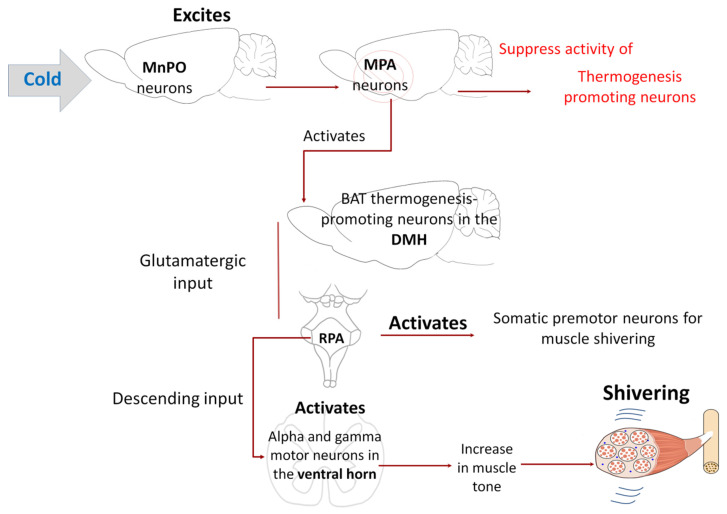
Neurophysiological mechanism for developing the shivering thermogenesis response. This mechanism is quite similar to the one without shivering; the difference lies in the fact that the activated neurons of the raphe pallidus area (RPA) are the alpha/gamma motor neurons in the ventral horn of the spinal cord. Activation of gamma motor neurons is involved in muscle tone, an event that precedes the development of muscular shivering [54]. Median preoptic nucleus (MnPO); medial preoptic area (MPA); dorsomedial hypothalamic nucleus (DMH).

Shivering refers to rapid, repetitive muscle contractions that generate heat when an organism has a fever or is exposed to cold temperatures. Tan and Knight [8] noted that the regulation of shivering also involves a group of structures that govern other physiological responses. Observations from decades ago showed that direct cooling of the POA fostered shivering induced by environmental cold in goats (*Capra aegagrus hircus*) [131] and canines (*Canis lupus familiaris*) [132] while stimulating the DMH, rRPA, and adjacent structures increased cold-induced shivering, and PGE2 in both rats [2] and cats (*Felis catus*) [133]. Like BAT thermogenesis, the shivering circuit requires activation of the thermogenesis-promoting neurons in the DMH and RPa. According to Nakamura and Morrison [2], cold activates the shivering-promoting neurons of the DMH by eliminating tonically active inhibitory input from the POA. Subsequently, those neurons activate the somatic premotor neurons for muscle shivering found in the RPa. Contrary to what was observed in the neural circuit for the sympathetic control of BAT, where the RPa neurons activate sympathetic preganglionic neurons in the intermediolateral cell column (IML), in this case, the descending input from the RPa for shivering activates alpha and gamma motor neurons [134] in the ventral horn of the spinal cord of rats (Figure 9). Interestingly, the activation of gamma motor neurons can increase the muscle tone preceding shivering and augmenting the intensity and threshold of the shivering response [54].

Although various studies have demonstrated the existence of direct descending projections from RPa neurons towards ventral horn somatomotor neurons, the pathways and mechanisms through which the alpha/gamma motor neurons are activated during shivering are not yet fully understood [8,54].

## 6. Thermoregulating Behavior in Mammals

**Figure 10:**
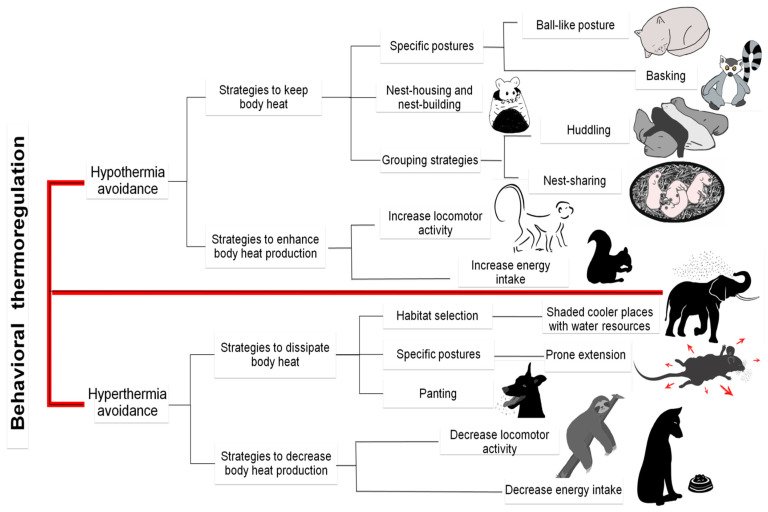
Behavior strategies used to control body heat. These are divided into those performed to prevent (i) hypothermia and (ii) hyperthermia. It is important to note that such factors as the time of day, season, gender, and age, can affect these thermoregulatory adjustments [7]. (Exclusive images created by Lupita Mota R.).

Animals are also capable of performing voluntary behaviors that alter their local thermal environment. These thermoregulating behaviors are carried out by endotherms (mammals and birds), reptiles, fish, and a high percentage of invertebrates that depend almost exclusively on behavioral elements to respond to external temperature changes. In addition to their antiquity, thermoregulating behaviors—at least those in mammals—are of the “motivated” type; that is, they occur in response to a motivation. For this reason, the temperature can be used as a reward to train animals to perform specific tasks, as in rats exposed to cold, who learn to press a button to turn on a heat lamp [14,15]. The above suggests that thermoregulating behaviors are driven by the same motivational system that preserves other behaviors—eating and drinking, for example—that arise in response to homeostatic needs [8]. Thermoregulating behaviors are divided into (i) those performed to prevent hypothermia and (ii) those carried out to prevent hyperthermia (Figure 10). The former includes strategies to conserve body heat, such as adopting certain postures (ball-like position) or basking under the sun, and nest-building, huddling, and nest-sharing. Other behaviors improve heat generation, for example, increasing locomotor activity and energy intake. Behaviors for preventing hyperthermia, in contrast, are based on strategies that dissipate heat, such as cold-seeking, shade-seeking, specific postures (exposure to wind, prone position/body extension, posting on rocks), moving the ears—in elephants [135,136]—and panting. Furthermore, actions that decrease heat generation, including lowering energy intake and locomotor activity [7]. Mammals perform these behaviors when exposed to severe thermal conditions in which their autonomous regulation loses effectiveness, and valuable body resources like water and energy could be compromised. In humans—and perhaps some other mammals—these behaviors focus on avoiding thermal discomfort [137]. Thus, requiring participation by emotion-related regions of the cerebral cortex and amygdala [138] activated by innocuous cutaneous thermal stimuli [1,139].

Almeida et al. [140] and Tan and Knight [8] maintain that the neural circuitry involved in behavioral responses is still poorly understood. One mechanism in rodents—animals that cannot sweat—consists of spreading their saliva over the skin surface to lose heat through evaporation. Salivation can be elicited by increasing the temperature of the anterior hypothalamus [141], as is seen in panting in mammals like cats (*Felis catus*) [142]. However, the detailed pathways leading from the anterior hypothalamus to the salivary nucleus are still unclear. Although thermal salivation decreases when lesions occur to the anterior [143], lateral [144], and ventromedial hypothalamus [145], it is believed that the simplest pathway is the one through which the preoptic area of the hypothalamus (POA) activates lateral hypothalamic inputs to the superior salivary nucleus [146]. However, grooming activity can be induced by increasing the temperature of the posterior—not anterior—hypothalamus, suggesting that some behavioral thermoregulating responses may be triggered in areas distinct from the POA [147]. Furthermore, the increase of warmth-induced locomotor activity that appears in rats upon stimulating the septal area, ventral midbrain, and dorsal medulla has not been affected after lesions in either these areas or in the POA [148,149]. In contrast, studies with rats have shown that activating the POA with heat induces prone extension behavior [148], while lesions in this region, and the ventral part of the MnPO, reduce or eliminate this behavior [149,150].

The evidence suggests that local cooling or heating of the POA is sufficient but not necessary for activating most thermoregulatory behaviors that reverse changes in the core body temperature [1]. Observations have shown that optogenetic stimulation of POA^PACAP/BDNF^ neurons activated by heat induces cold-seeking but inhibits nest-building [106]. Chemogenetic stimulation of POA^LepR^ neurons induces prone extension behavior to dissipate heat in mice [72]. Similarly, autonomous and behavioral responses to manipulations of the POA can stimulate thermoregulating behaviors, likely through the dorsomedial hypothalamic nucleus (DMH) [151].

Several authors mention that lesions that ablate the POA in rats leave most thermoregulating behaviors intact [149,151,152]. Carlisle [152] observed that Sprague-Dawley rats held in a cold environment after lesions to the POA improved their responses to the heat-reinforced operant procedure to which they were subjected. Probably to compensate for their loss of autonomous thermoregulation.

Most lesioning experiments have been unsuccessful in identifying the forebrain region necessary for thermoregulating behaviors, as the POA is for autonomous responses. Observations of primates have shown that the thalamus plays an important role in temperature perception [153,154]. However, recent studies with rodents have demonstrated that even after this area is injured, no alterations are detected in behaviors related to thermoregulation [63].

Other authors have pointed out that rats may require the POA for prone-extension behavior induced by warmth [149], the dorsomedial hypothalamus (DMH) for cold-seeking behavior induced by systematic inflammation [151,155], and the MnPO for the cold-seeking response to systemic salt-loading [156]. The failure to block thermoregulating behaviors by injuring the POA has led some researchers to conclude that this area is not involved in those specific responses [140]. Nevertheless, this is hardly possible considering all the evidence indicating that stimulation of the POA suffices to orchestrate various thermoregulating behaviors. According to Tan and Knight [8], these conflicting results could be explained by the complexity of the POA circuit, which contains many different intermingled cell types that make it difficult to interpret the results of lesioning experiments lacking adequate cell-type specificity. This proposal is based on observations of lesions in the hypothalamic arcuate nucleus (ARC). When not specific, those injuries cause hyperphagia and obesity in rats, suggesting that the ARC functions as an anxiety center [157], while ablation of a specific type of ARC cells (AgRP neurons) causes starvation in mice [158]. Thus, we hope that future research will focus on manipulations of specific cell types to re-analyze the role of the POA and downstream structures in controlling thermoregulating behaviors.

## 7. Areas of Opportunity and Practical Applications

The study of the hypothalamic neuromodulation of thermoregulation has broad areas of opportunity with practical applications strengthened by the availability of valuable tools like IRT. Some of these areas of opportunity and application could be: understanding the effect of climate change on behavior, productivity, mating, and partner-searching in numerous species; detecting the effects of exercise on animals involved in sporting activities; identifying microvascular changes that occur in response to fear, pleasure, pain, and other situations that cause stress in animals; and advancing the study of thermoregulating behaviors. Research of this kind could, in turn, help develop potential solutions to existing problems related to the drastic modification of environments that might induce lack of appetite, low productivity, neonatal hypothermia, and thermal shock, among others. Studies of the skin concerning temperature modulation have been based mainly on the IRT technique. However, numerous characteristics must be considered before we can be sure that the skin’s surface temperature plays an important role in transmitting thermal stimuli while acting in concert with the core body temperature.

Scientific evidence indicates that these physiological and behavioral mechanisms share similar neurophysiological pathways (i.e., the thermal signals that arrive at the spinal cord before moving towards the POA, distributed to distinct neuronal circuits to generate efferent responses) allow them to respond. Some of the neurophysiological aspects involved in thermoregulation require further study, especially the efferent pathways implicated. This is because the exact pathways allowing the cerebral cortex to transmit signals to specific brainstem regions are not yet known. There is still controversy over the participation of the POA in the development of behavioral responses. Thus, we expect future research to focus on manipulating specific cell types to identify the cells involved in developing autonomous responses, differentiating those that respond specifically to cold or heat stimuli. Another key objective is to understand the nervous system connections that organize thermoregulating behaviors in homeotherm and ectotherm organisms, establishing models of nervous systems that explain the changes observed. Of course, we must be aware that this is an enormously challenging task because we are striving to understand complex mechanisms.

## 8. Conclusions

Mammals use several mechanisms to maintain thermal homeostasis. Some require using valuable resources, such as glucose or water to increase survival chances. Temperature regulation in mammals is highly organized to respond to different stimuli that can generate thermal changes: the environment, reproduction stages, nutritional and diet status, and even the inflammatory processes affecting them. In this regard, the neurophysiological pathways that have been mentioned herein reveal a complex system intimately intertwined with other systems. The mechanisms associated with thermal homeostasis involve the skin, which is an organ of crucial importance in responses to thermal stimuli because it is part of the first line of the detection of thermal changes and microcirculation, participating in vasomotor processes as a mediating factor of changes in blood flow that permit the dissipation or retention of heat through vasodilatation and vasoconstriction. However, the participation and integrity of anatomical regions like the POA, cerebral cortex, afferent nerves, and spinal cord are essential for the adequate development of these thermoregulating responses, as they regulate both physiological and behavioral responses.

Current knowledge of the physiological processes and complex anatomical structures, like the nervous systems and their close relation to mechanisms of thermoregulation, is still limited. We cannot explain processes such as behavioral changes, pathways, or connections that trigger mechanisms like vasodilatation and panting, or the connection between emotions and thermoregulation from results in this evolutionary neuroscience of thermoregulation. An increasingly precise understanding of thermoregulation will allow us to design and apply practical methods in the field, like in zootechnics and clinical medicine, without compromising animal welfare. The results obtained should bring improvements in the production, quality of life, and survival of animals.

## Data Availability

Not Applicable.

## Abbreviations

POA: preoptic area; ARC: arcuate nucleus of the hypothalamus; BAT: brown adipose tissue; BDNF: brain-derived neurotrophic factor; CNS: central nervous system; CVC: cutaneous vasoconstriction; DFD: dark, firm, and dry meat; DMH: dorsomedial hypothalamus; IML: intermediolateral column; IRT: infrared thermography; LH: lateral hypothalamus; LepR: leptin receptors; LPB: lateral parabrachial nucleus; LPBel: external lateral parabrachial nucleus; MnPO: median preoptic nucleus; MPO: medial preoptic area; PAG: periaqueductal gray; PGD2: prostaglandin D2; PSE: pale, soft exudative meat; RPa: raphe pallidus area; RPA: raphe pallidus; rRPA: rostral raphe pallidus; RVLM: rostral ventrolateral medulla; RVMM: rostral ventromedial medulla; SNS: sympathetic nervous system; TRP: transient receptor potential; TRPM2: transient thermosensitive reception channels; vMAP: medial ventral preoptic area; vLPO: ventrolateral preoptic area; UCP1: uncoupling protein-1; VTA: ventral tegmental area; WHC: water-holding capacity.
